# The association between serum vitamin D status and dental caries or molar incisor hypomineralisation in 7–9-year-old Norwegian children: a cross-sectional study

**DOI:** 10.1186/s12889-024-17745-1

**Published:** 2024-01-22

**Authors:** Torunn Børsting, Tone Natland Fagerhaug, Annemarie Schuller, Paula van Dommelen, Signe Nilssen Stafne, Siv Mørkved, Astrid Kamilla Stunes, Miriam K. Gustafsson, Unni Syversen, Yi-Qian Sun, Marit S. Skeie

**Affiliations:** 1 Center for Oral Health Services and Research, Mid-Norway (TkMidt), Trondheim, Norway; 2 Department of Public Health and Nursing, Norwegian University of Science and Technology (NTNU), Trondheim, Norway; 3 Department of Child Health, the Netherlands Organization for Applied Scientific Research (TNO), Leiden, The Netherlands; 4 Centre of Dentistry and Oral Hygiene, University Medical Center Groningen, University of Groningen, Groningen, The Netherlands; 5 Department of Clinical Service, Trondheim University Hospital (St. Olavs Hospital), Trondheim, Norway; 6 Department of Clinical and Molecular Medicine, Norwegian University of Science and Technology (NTNU), Trondheim, Norway; 7 Medical Clinic, Trondheim University Hospital (St Olavs Hospital), Trondheim, Norway; 8 Department of Endocrinology, Trondheim University Hospital (St. Olavs Hospital), Trondheim, Norway; 9 Regional Education Center (RegUt), Helse Midt-Norge, Trondheim, Norway; 10 Department of Clinical Dentistry, University of Bergen, Bergen, Norway

**Keywords:** Oral health, Dental caries, Molar incisor hypomineralisation, MIH, Vitamin D, Child health, Pedodontics, Public health, Dental public health

## Abstract

**Background:**

Research focusing on the association between serum vitamin D and oral health outcomes in children, such as dental caries and molar incisor hypomineralisation (MIH), shows inconsistent results. Previous studies have predominantly investigated dental caries and MIH as dichotomized outcomes, which limits the information on their distribution. In addition, the methods used for analysing serum vitamin D have varied. The present study aimed to investigate potential associations between serum vitamin D status measured by Liquid Chromatography with Tandem Mass Spectrometry (LC-MS/MS) and the prevalence, as well as the number of teeth, affected by dental caries or MIH among 7–9-year-old Norwegian children.

**Methods:**

The study had a cross-sectional design and included 101 children aged 7–9 years. Serum 25-hydroxyvitamin D (25(OH)D) was measured and included as continuous (per 25 nmol/l) and categorised (insufficient (< 50 nmol/l) and sufficient (≥50 nmol/l)) exposure variables. Adjusted negative binomial hurdle models were used to investigate the potential associations between serum vitamin D and the oral health outcomes (dental caries and MIH) adjusted for sex, age, body mass index, season of blood draw, and mother’s educational level.

**Results:**

Of the 101 children in the total sample, 27% had insufficient vitamin D levels (< 50 nmol/l). The descriptive analysis indicated that the children with insufficient vitamin D levels had a higher prevalence (33.3%) and a higher number of teeth affected by dental caries (mean (SD) = 0.7 (1.4)), compared to children with sufficient levels of vitamin D (21.6% and mean (SD) = 0.4 (0.8), respectively). The same holds for MIH, with a higher prevalence (38.5%) and a higher number of teeth affected (mean (SD) = 1.2 (2.3)), compared to children with sufficient levels of vitamin D (30.1% and mean (SD) = 0.8 (1.6), respectively). However, in the adjusted hurdle model analysis, neither the prevalence or number of teeth affected by caries or MIH showed statistically significant associations with having insufficient or lower vitamin D levels.

**Conclusions:**

Vitamin D status was not significantly associated with the prevalence and number of teeth affected by caries and MIH among the participating children. Large prospective studies with multiple serum vitamin D measurements and oral examinations throughout childhood are warranted to elucidate the relationship.

## Background

Vitamin D is a steroid hormone with multiple biological functions, of which a central function is to stimulate the intestinal absorption of calcium and phosphate to ensure normal levels in the blood. This is important to facilitate adequate mineralisation of hard tissues such as bones and teeth. The main source of vitamin D is exposure to ultraviolet (UV) light, but it can also be absorbed through the intake of food or supplements containing vitamin D [1]. Vitamin D is metabolised in the liver to its main circulating form, 25-hydroxyvitamin D (25(OH)D). Further, 25(OH)D can be metabolised into 1,25-dihydroxyvitamin D (1,25(OH)2D), the biologically active form of vitamin D, which interacts with the vitamin D receptor. Expression of the vitamin D receptor has been found in several tissues throughout the body, suggesting that vitamin D has additional influence on many biological processes beyond regulating the calcium and phosphate metabolism, including modulation of the immune system [2–4].

There are several mechanisms by which vitamin D may have a protective role in oral health. Vitamin D is important in ensuring appropriate tooth development and mineralisation through its regulation of calcium and phosphate absorption, but also potentially by activating vitamin D receptors that influence the tooth mineralisation processes [1]. Vitamin D receptors have, for example, been found in both ameloblasts and odontoblasts [5]. Another proposed mechanism is that vitamin D may modulate the immune response to caries by inducing antimicrobial peptides, or other inflammatory markers, as a response to the bacterial infection [1].

During the last decade, there have been several studies investigating the relationship between serum vitamin D and dental caries in children [6–14]. Although the results have been somewhat inconsistent, a recent systematic review and meta-analysis, which included many of these studies, found a pooled relative risk of 1.22 (95% CI 1.18–1.25) between vitamin D deficiency (< 50 nmol/l) and dental caries in children (dmft/DMFT) [15]. Fewer studies have been conducted among children to investigate potential associations between serum vitamin D and molar incisor hypomineralisation (MIH), a developmental defect of enamel, and these studies have also shown inconsistent results [10, 16].

Most of these studies have dichotomised the oral health outcomes (dental caries and/or MIH). However, only expressing prevalence when describing an oral disease or condition, limits the information on their distribution. Including continuous variables in the analysis provides a better picture of the children with more than one affected tooth. So far, studies on vitamin D that includes oral health outcomes as continuous variables in adjusted regression analyses have been sporadic. As far as we are aware, only one of the studies referred to above has done so [10]. Moreover, there is a variation in the analysis methods used for measuring serum 25(OH)D, and many previous studies have used immunoassays [9–11]. Currently, Liquid Chromatography with Tandem Mass Spectrometry (LC-MS/MS) is regarded as the gold standard, and it has been shown that immunoassays underestimate the serum 25(OH)D concentration compared to that measured by LC-MS/MS [17, 18].

Furthermore, it is of interest to acquire more knowledge about the potential impact of vitamin D on oral health among those living in far-north countries like Norway, where it is not possible to obtain vitamin D through sunlight for a large portion of the year. A recent study from Norway has shown an association between maternal vitamin D in pregnancy and the number of teeth affected by MIH in the offspring [19]. However as far as we are aware, studies with a focus on the relationship between vitamin D and oral health in the general Norwegian child population are lacking.

Therefore, the current study aimed to investigate the potential associations between serum vitamin D status measured by LC-MS/MS and the prevalence, as well as the number of teeth, affected by dental caries or MIH among 7–9-year-old Norwegian children. Our hypothesis is that the children with the lowest serum vitamin D levels would be the most affected by dental caries and MIH.

## Methods

### Study design, setting, and participants

The current study has a cross-sectional design. Data was collected as part of the TRaining In Pregnancy study (TRIP-study), which initially was a randomized controlled trial (RCT) investigating the effect of a pregnancy exercise programme on gestational diabetes (further details can be found in Stafne, Salvesen [20]). Women were recruited to the study at their routine second trimester ultrasound and the eligibility criteria for inclusion were age ≥ 18 and having a singleton pregnancy. Those with high-risk pregnancies or other diseases, which could interfere with participation, were excluded. Seven years later, a follow-up of the mothers included in the RCT and their offspring was conducted. As part of the 7-year follow-up, the children were invited to participate in an oral health sub-study (TRIP-tann), which included an oral examination. In addition, the children living in Trondheim were invited to another sub-study on bone health (TRIP-bein), which included the collection of a serum sample. The oral examinations were conducted at the Centers for Oral Health Services and Research in Trondheim and Stavanger, Norway, between May 2016 and August 2017, when the children were 7–9 years of age. The serum samples, collected as part of the TRIP-bein sub-study, were taken at Trondheim University Hospital between November 2016 and December 2018.

### Assessment of child vitamin D status

Overnight fasting blood samples were taken in the morning (between 07:30 and 09:00). The blood samples were collected by standard venipuncture, collected in vacuum tubes, and sat for 30 minutes at room temperature. The samples were then centrifuged (3000 g/4 °C/10 minutes) before serum was aliquoted and stored at − 80 °C until further analyses. In March 2021, the serum from all participants was analysed simultaneously for 25(OH)D by an accredited Liquid Chromatography with Tandem Mass Spectrometry (LC-MS/MS) method with DEQAS quality control (www.deqas.org). In addition, serum intact parathyroid hormone (PTH) was analysed by an accredited non-competitive immunoluminometric assay (ILMA) with an Immulite 2000 Intact PTH Kit (Siemens Cat# L2KPP-15, RRID: AB_2782968s). Both serum analyses were performed at the Hormone Laboratory, Oslo University Hospital, Oslo, Norway.

The children’s serum 25(OH)D levels were used as the exposure variable in the current study to assess vitamin D status. Reflecting both vitamin D intake and UV exposure, 25(OH)D has been recognised as the best parameter to evaluate vitamin D status [21]. For the analyses, we included serum 25(OH)D as both a continuous exposure (per 25 nmol/l) and a binary exposure categorised as insufficient (serum 25(OH)D < 50 nmol/l) and sufficient (serum 25(OH)D ≥ 50 nmol/l). The categorisation was according to current nutritional guidelines [22, 23]. As part of the background variables, we also assessed whether the PTH levels of the children were in the normal range [24]. Along with vitamin D, PTH indicates whether the calcium metabolism is functioning normally.

### Oral examination

The main focus of the oral examination was to assess caries and MIH, and was conducted by two calibrated dentists experienced in paediatric dentistry, who were blinded to the vitamin D status of the children. Descriptions of the calibration training and results have previously been published [19, 25]. The caries collection was based on the dmft/DMFT index (decayed, missing, and filled primary/permanent teeth) using a detailed 5-graded caries diagnostic tool [26]. Grades 1–2 denoted enamel caries (d_1–2_/D_1–2_) and grades 3–5 dentine caries (d_3–5_/D d_3–5_). Due to the low caries prevalence in the study population, caries in the primary and permanent dentition were combined into one variable for the statistical analysis. Caries prevalence was therefore defined as having at least one primary or permanent tooth with caries experience at the enamel or dentine level (d_1-5_mft/D_1-5_MFT > 0), while mean caries experience included the total d_1-5_mft/D_1-5_MFT for each child. The examiners were instructed on how to distinguish between missing due to caries and missing due to exfoliation.

MIH was recorded according to the MIH-index [27], which has been recommended for epidemiological studies in a recently published best practice guideline for MIH [28]. Registrations were made if 1/3 of the tooth or more had erupted. Hypomineralisation defects were recorded if they were 1 mm or larger. Defects on approximal surfaces and diffuse opacities were not included. The colour of the hypomineralisation defects (white/cream to yellow/brown), post-eruptive enamel breakdown, atypical caries, or atypical fillings, were recorded. Prevalence of MIH was defined as having at least one first permanent molar with MIH. The number of teeth affected by MIH included all affected first permanent molars and, according to the guidelines, all affected permanent incisors were also included, if a first permanent molar was simultaneously affected.

### Potential confounding factors

Potential confounding factors were acquired from the TRIP main- and sub-study records while the sex of the child and date of birth were acquired from their birth records. Age at the oral exam was calculated from the date of birth and the date of the dental exam (continuously in years). The height and weight of the child were measured in the TRIP bone health sub-study and used to calculate the children’s body mass index (BMI) (weight (kg) divided by the squared value of height (m2)). BMI scores were then categorised into thinness, normal weight, and overweight according to the iso-BMI standard, which is based on the International Obesity Task Force (IOTF) definitions and used as the reference values for the growth charts used by health services in Norway [29]. The continuous BMI variable was used in the analysis, as there were few children in the thinness and overweight categories. The season of blood draw for the child blood samples was initially categorised into four categories, the same used in the TRIP-study. However, as there were very few who participated during the summer months (*n* = 3), we chose to combine the variable into two categories for analysis purposes: winter or spring (December–May); and summer or autumn (June–November). The mother’s educational level was obtained from the baseline data collection of the TRIP-study, and was categorised into those who had high school or university education ≤4 years and those who had university education > 4 years. All the mothers and participating children were of Norwegian origin and had a fair skin-tone.

### Statistical analyses

The descriptive analysis in the current study was performed using Stata SE version 16.1 (StataCorp LLC, TX, USA) and presented as frequency and percent (%) for categorical variables, and mean and standard deviation (SD) for continuous variables. Hurdle models were used to analyse potential associations between child vitamin D status and oral health outcomes using R version 4.1.2 (R Core Team 2021) and the R package ‘pscl’ [30]. This type of regression analysis accounts for positively skewed distributions with a large number of zero values, which is often the case with oral health data where outcomes have a relatively high number of non-affected teeth [31]. The data is analysed in two parts, first by performing logistic regression to assess the prevalence of the outcome according to the exposure (the zero model), and second by performing negative binomial regression to assess the number of affected teeth among those affected in the exposed and unexposed groups (the count model). The estimates calculated from the logistic regression analysis are presented as odds ratios (ORs) and 95% confidence intervals (95% CIs), while the estimates from the negative binomial analysis are presented as rate ratios (RRs) and 95% CIs. In addition to the 95% CIs, *p* values were included to assess statistical significance, with a *p* value < 0.05 (two-sided) considered statistically significant. In the adjusted analysis, we included available health and demographic variables, which based on prior knowledge, could be potential confounders. The included factors were child sex, age, BMI, the season of blood draw, and the mother’s educational level. Two participants were missing from the MIH variable, due to no erupted first permanent molars, and they were excluded from the relevant analyses.

## Results

**Fig. 1:**
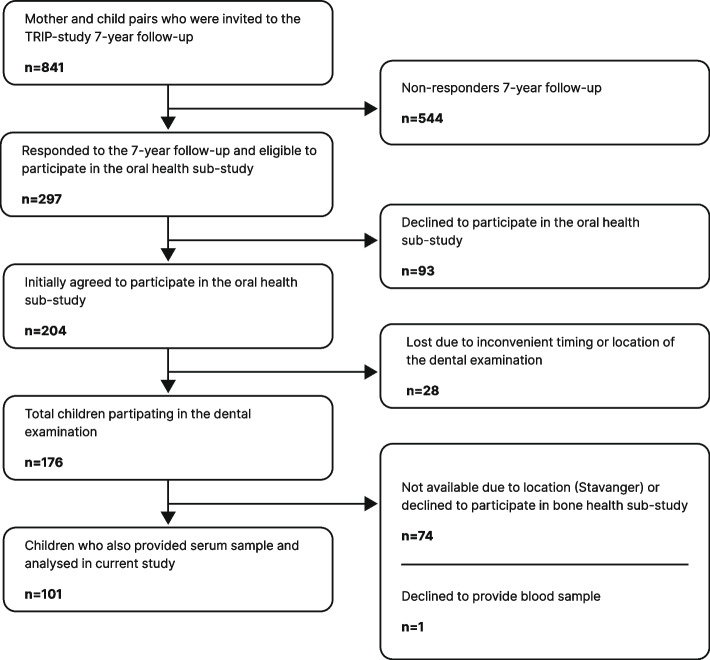
Flow chart of study recruitment

Figure 1 shows the recruitment of participants to the current study. Of the 176 children who participated in the oral examination as part of the TRIP-tann sub-study, 101 (57%) also participated in the TRIP-bein sub-study and agreed/were able to provide a serum sample, and were included in the analyses for the present study. The mean age of the participants was 8.1 (SD 0.35), and 53% were girls (Table 1). Of the 101 participants in the total sample, 27% had insufficient vitamin D levels. The average serum PTH levels of the children were in the normal range. The descriptive analysis indicated that there was a higher proportion with insufficient vitamin D levels among the girls, those who had their blood samples taken in winter or spring, and those who had a mother in the category representing the lowest educational levels (Table 1).

**Table 1:** Child health and demographic characteristics by serum vitamin D status

	Total participants (*n* = 101)	Child hydroxyvitamin D (25(OH)D)	
		Insufficient < 50 nmol/l (*n* = 27)	Sufficient ≥50 nmol/l (*n* = 74)
**Child characteristics**			
Age at oral exam, mean (SD)	8.1 (0.4)	8.1 (0.4)	8.1 (0.4)
Sex, n (%)			
Boys	48 (47.5)	11 (40.7)	37 (50.0)
Girls	53 (52.5)	16 (59.3)	37 (50.0)
BMI, mean (SD)	16.5 (1.7)	16.3 (1.3)	16.6 (1.8)
iso-BMI, n (%)			
Thinness	3 (3.0)	1 (3.7)	2 (2.7)
Normal weight	86 (85.1)	24 (88.9)	62 (83.8)
Overweight	12 (11.9)	2 (7.4)	10 (13.5)
Season of blood draw, n (%)			
Winter or spring (Dec - May)	66 (65.4)	23 (85.2)	43 (58.1)
Summer or autumn (June - Nov)	35 (34.6)	4 (14.8)	31 (41.9)
25OHD nmol/l, mean (SD)	59.2 (15.7)	40.6 (8.4)	66.0 (11.7)
PTH pmol/l^a^, mean (SD)	2.42 (1.1)	2.56 (1.1)	2.37 (1.1)
Mother’s educational level, n (%)			
High school or university ≤4 years	41 (40.6)	14 (51.9)	27 (36.5)
University > 4 years	60 (59.4)	13 (48.1)	47 (63.5)

^a^1 missing due to PTH being under the level of detection

Among the 101 participants, the caries prevalence with enamel caries included (d_1-5_mft/D_1-5_MFT) was 25%, while the MIH prevalence was 32% (Table 2). The caries prevalence at the dentine level (d_3–5_mft/D_3–5_MFT > 0), enamel caries excluded, was 15%, while the prevalence of a yellow/brown opacity on an MIH-tooth was 8%. There was a higher proportion of children affected by caries or MIH in the insufficient compared to the sufficient vitamin D group (+ 11.7% and + 8.4%, respectively), and those in the insufficient group also had a higher mean caries experience (+ 0.3) and mean number of teeth affected with MIH (+ 0.4) (Table 2).

**Table 2:** Child oral health outcomes by serum vitamin D status

	Total participants (*n* = 101)	Child hydroxyvitamin D (25(OH)D)	
		Insufficient < 50 nmol/l (*n* = 27)	Sufficient ≥50 nmol/l (*n* = 74)
**Child oral health outcomes**			
Caries			
Caries prevalence (d_1-5_mft/D_1-5_MFT > 0), n (%)	25 (24.8)	9 (33.3)	16 (21.6)
Caries experience (d_1-5_mft/D_1-5_MFT), mean (SD)	0.5 (1.0)	0.7 (1.4)	0.4 (0.8)
Dentine caries prevalence (d_3–5_mft/D_3–5_MFT > 0), n (%)	15 (14.9)	5 (18.5)	10 (13.5)
Dentine caries experience (d_3–5_mft/D_3–5_MFT), mean (SD)	0.3 (0.8)	0.5 (1.3)	0.2 (0.6)
Enamel hypomineralisation			
MIH^a^ prevalence (> 0), n (%)	32 (32.3)	10 (38.5)	22 (30.1)
MIH^a^, mean (SD)	0.9 (1.8)	1.2 (2.3)	0.8 (1.6)
MIH^a^ yellow/brown opacities prevalence (> 0), n (%)	8 (8.1)	4 (15.4)	4 (5.5)
MIH^a^ yellow/brown opacities, mean (SD)	0.1 (0.5)	0.2 (0.5)	0.1 (0.5)

^a^MIH, 2 missing - no erupted first permanent molars. The MIH count variable includes affected incisors in cases with at least one affected first permanent molar as well

In the hurdle analysis (Table 3), the adjusted estimates for both caries prevalence (including enamel caries) and MIH prevalence, i.e. the zero model, indicated higher odds of having caries or MIH among those with insufficient vitamin D, however, none of the estimates reached statistical significance and the confidence intervals were wide. For the count model, the adjusted estimate for caries experience (only including those affected), was close to null when vitamin D was included as a categorical variable, indicating no difference between the insufficient and sufficient vitamin D groups. However, when vitamin D was included as a continuous variable, the estimate indicated a higher risk of caries with decreasing vitamin D. The finding was close to statistically significant, but the 95% CI was very wide. For MIH, the estimates indicated that those with insufficient/lower vitamin D levels had a lower number of teeth with MIH, but again the estimates did not reach statistical significance and the confidence intervals were wide.

**Table 3:** Regression analyses with child 25(OH)D levels and oral health outcomes

	Oral health outcomes prevalence (zero model)				Oral health outcomes extent (count model)			
	Unadjusted OR (95% CI)	*p*-value	Adjusted OR (95% CI)*	*p*-value	Unadjusted RR (95% CI)	*p*-value	Adjusted RR (95% CI)*	*p*-value
Caries experience primary and permanent teeth (d_1-5_mft/D_1-5_MFT)								
Insufficient (< 50 nmol/l)	1.81 (0.69–4.80)	*p* = 0.23	1.94 (0.67–5.61)	*p* = 0.23	1.49 (0.44–5.03)	*p* = 0.52	1.01 (0.14–7.06)	*p* = 0.99
Sufficient (≥50 nmol/l)	Ref		Ref		Ref		Ref	
Continuous 25(OH)D (per 25 unit decrease)	1.10 (0.53–2.27)	*p* = 0.80	1.15 (0.53–2.50)	*p* = 0.72	2.22 (0.96–5.26)	*p* = 0.06	2.94 (0.73–12.5)	*p* = 0.13
MIH**								
Insufficient (< 50 nmol/l)	1.45 (0.57–3.69)	*p* = 0.44	1.19 (0.43–3.29)	*p* = 0.74	1.24 (0.55–2.76)	*p* = 0.61	0.81 (0.33–1.97)	*p* = 0.64
Sufficient (≥50 nmol/l)	Ref		Ref		Ref		Ref	
Continuous 25(OH)D (per 25 unit decrease)	1.45 (0.74–2.86)	*p* = 0.28	1.45 (0.70–2.94)	*p* = 0.32	0.93 (0.51–1.69)	*p* = 0.82	0.68 (0.34–1.33)	*p* = 0.26

* Model 1 adjusted for child sex (cat.), age (cont.), BMI (cont.), season of blood draw (cat.), and mother’s education (cat.)

** 2 participants had no erupted MIH index teeth and were therefore not included in the regression analysis for the MIH outcome. Two participants had two erupted MIH-index teeth, and one participant had one erupted MIH-index tooth, and these participants were still included in the regression analysis. Affected incisors are included in cases with at least one affected first permanent molar as well

## Discussion

In this study, we investigated potential associations between serum vitamin D levels and caries or MIH among Norwegian children between 7 and 9 years of age. The descriptive analysis indicated that the children with insufficient vitamin D levels (< 50 nmol/l) had a higher prevalence and a higher number of teeth affected by caries and MIH, compared to children with sufficient levels of vitamin D. However, in the hurdle model analysis, none of the results showed statistically significant associations with having insufficient/lower vitamin D levels, which was contrary to our hypothesis. The wide confidence intervals for most of the estimates suggest that the study may have been underpowered.

Among previous studies that included children of a similar age and the same cut-off for insufficient vitamin D (< 50 nmol/l) as in our study, one of the studies found no association with the prevalence of caries in the permanent dentition of 10–12-year-olds [11], while another study found a significant association with the prevalence of caries in children examined between 6 and 10 years of age (mixed dentition) [6]. In studies where vitamin D was included as a continuous variable, two studies found a weak inverse association (95% CIs just crossing one) between vitamin D status and the prevalence of dental caries in 8-year-olds [12] and 5–9-year-olds [7]. The study that did not find a significant association [11], was the only study that used an immunoassay to measure the serum 25(OH)D level in the children, which may explain the discrepancy compared to the other studies.

Concerning MIH, one previous study found no association between serum vitamin D status and the prevalence of MIH at 6 years of age [16]. Another previous study used hurdle models, as in our study, to investigate the association between serum vitamin D and both dental caries and MIH in 10-year-olds [10]. Consistently with our study, they did not find an association between those with higher serum vitamin D levels (measured continuously) and the prevalence of dental caries, but they did find a significant association with dentine caries experience (i.e. number of affected teeth). In addition, they found significant associations between those with higher serum vitamin D levels and both the prevalence and number of teeth affected by MIH. A limitation of this latter study is that it used an immunoassay to analyse serum 25(OH)D levels in the children, however, it was the only other study to include caries and MIH as both dichotomous and continuous outcomes in their statistical analysis [10].

One potential explanation for the lack of significant associations in our study is that the children in the sample had a low caries prevalence and caries experience when compared to other studies of similar populations. For caries experience at the dentine level, for example, only 15% of the 7–9-year-olds in our study had at least one affected tooth and the mean d_3–5_mft/D_3–5_MFT for the whole sample was 0.28 (SD 0.84). Compared to this, a 2010 study from Denmark, found that among 7-year-olds, 51% had at least one tooth with caries experience at the dentine level and a mean of 4.1 affected tooth surfaces (dmfs/DMFS) [32]. This previous study included families with more varied socio-economic and socio-cultural backgrounds than in our study and found that the mother’s education, immigrant background, and family income were associated with caries prevalence. The reasonably high educational level and non-immigrant background of the mothers in our study could therefore partly explain why fewer children were affected by caries.

Furthermore, previous studies have shown that immigrant background and socioeconomic status are also associated with vitamin D status and that Nordic countries, despite the northern latitude, have been found to have a lower prevalence of vitamin D deficiency than other European countries, most likely due to higher use of vitamin D supplements and food fortification [33]. Therefore, the background characteristics of our sample may have also contributed to the reasonably high average vitamin D status of the participating children. Thus, future studies should ensure the inclusion of participants/populations with a higher risk of caries and/or vitamin D insufficiency/deficiency.

Regarding MIH, an issue to consider is that the children were examined at an age where the MIH teeth have erupted, and thus, a majority of the enamel mineralisation has already taken place [34]. Although the MIH teeth were examined at the optimal age [35], i.e. newly erupted, it could be argued that the assessment of serum vitamin D should have been done at a younger age when the enamel of the MIH teeth was at an earlier developmental phase. However, vitamin D status in infancy has been found to be a predictor of vitamin D status in early school-age children [36], suggesting that vitamin D levels may stay reasonably consistent over time. Therefore, the vitamin D status measured at 7–9 years, as in the present study, could also reflect earlier vitamin D status. A previous study [10] found that even when vitamin D was measured at 10 years of age, there was an association with both the prevalence and number of teeth affected by MIH at the same age. However, in the future, conducting well-designed prospective studies with several serum vitamin D measurements and oral examinations throughout childhood would be an advantage to clarify the relationship.

Strengths of the current study included the availability of blood samples from the children, and that LC-MS/MS analysis was used to measure their serum 25(OH)D levels, which is considered the gold standard for analysing vitamin D metabolites in serum. In addition, two meticulously calibrated dentists experienced in paediatric dentistry, and also blinded to the children’s vitamin D status, performed the dental examinations. Furthermore, the analysis included investigating both prevalence and the extent (i.e. number of affected teeth) of the outcomes and was adjusted for several potential confounders. Finally, although affecting external generalisability, the homogeneity of the sample may have helped to reduce residual confounding.

The main limitation of the current study was the relatively low number of available participants who responded to both sub-studies. The loss of respondents may have introduced selection bias although in a previously published study, we showed that the sample population participating in the TRIP-tann oral health sub-study were similar to those in the original TRIP-study sample [19]. In addition, the smaller sample in the current study may have resulted in a lack of power to reach any potentially statistically significant associations, as was indicated by the wide confidence intervals. The count part of the hurdle model is particularly vulnerable to this, as only those who are affected by the outcome are included in this part of the analysis. Due to this, we cannot exclude that there are potential inverse associations between vitamin D status and the prevalence or number of teeth affected by dental caries and/or MIH. In addition, because of the low prevalence of the oral health outcomes and the number of teeth affected, we were not able to perform any sub-group analysis, for example, by only including those with caries at the dentine layer or those showing yellow/brown opacities on MIH teeth. Furthermore, due to the homogeneity of the sample (e.g. most of the children had a normal body weight, had well-educated mothers, and were of Norwegian origin), the current sample is not representative of the general child population in Norway. Finally, as the study used a cross-sectional design, the temporality between vitamin D level and development of MIH or dental caries could not be established.

## Conclusion

Vitamin D status was not significantly associated with the prevalence and number of teeth affected by caries and MIH among 7–9-year-old children in Norway. Large prospective studies with multiple serum vitamin D measurements and oral examinations throughout childhood are warranted to elucidate the relationship.

## Data Availability

The datasets analysed during the current study are available from the corresponding author on reasonable request.

## Abbreviations

dmft/DMFT: decayed, missing or filled primary (small letters) or permanent (capital letters) teeth
MIH: molar incisor hypomineralisation
25(OH)D: 25-hydroxyvitamin D
1,25(OH)2D: 1,25-dihydroxyvitamin D
PTH: parathyroid hormone
LC-MS/MS: liquid chromatography with tandem mass spectrometry
ILMA: immunoluminometric assay
BMI: body mass index
